# Editorial: Citizen Science and Social Innovation: Mutual Relations, Barriers, Needs, and Development Factors

**DOI:** 10.3389/fsoc.2022.836149

**Published:** 2022-02-10

**Authors:** Andrzej Klimczuk, Egle Butkeviciene, Minela Kerla

**Affiliations:** ^1^ Department of Public Policy, Collegium of Socio-Economics, SGH Warsaw School of Economics, Warsaw, Poland; ^2^ Faculty of Social Sciences, Arts and Humanities, Kaunas University of Technology, Kaunas, Lithuania; ^3^ The Association of Online Educators, Sarajevo, Bosnia and Herzegovina

**Keywords:** citizen science, co-creation, co-production, open science, participatory research, social innovation

## Overview

The presented Research Topic explores the potential of citizen science to contribute to the development of social innovations. It sets the ground for analysis of mutual relations between two strong and embedded in the literature concepts: citizen science and social innovation. Simultaneously, the collection opens a discussion on how these two ideas are intertwined, what are the significant barriers, and the need to use citizen science for social innovation.

As described by the Organisation for Economic Co-operation and Development and Eurostat (2018), social innovation refers to some new idea, new solution, or new design that makes a social impact in terms of conceptual, process, product, or organizational change, which aims to improve the lives of individuals and communities. This conceptual perspective lays a background for this Research Topic. It is possible to consider citizen science as social innovation. As emphasized by Butkeviciene et al. (2021), the relationship between citizen science and social innovation might be two-fold: citizen science as a novel practice might be considered as social innovation in the realm of the traditional research process, and citizen science might be treated as a vehicle to foster social innovation. These two approaches are present in theoretical debates and coherently intertwined in this collection. On the one hand, articles analyze methodological issues and the novelty of such methods as design thinking or action research. On the other hand, papers also investigate the factors such as translation specifics in citizen science, ecosystems of citizen science, or new learning environments that are supporting the development of social innovation.

The presented Research Topic includes seven articles prepared in total by 34 authors from the following countries: Australia, Austria, Czechia, Estonia, Finland, Germany, Ireland, Italy, Japan, Netherlands, Portugal, Singapore, Switzerland, and United Kingdom. Five journals were related to this Research Topic: “Frontiers in Sociology,” “Frontiers in Research Metrics and Analytics,” “Frontiers in Communication,” “Frontiers in Environmental Science,” and “Frontiers in Political Science.” This collection contains five types of articles covering: two original research articles (Goi and Tan; Heinisch), one perspective article (Roche et al.), two conceptual analysis articles (Eckhard et al.; Roche et al.), one review article (Scheibner et al.), and one methods article (Coulson et al.).

This Research Topic covers papers that critically evaluate the existing social innovations and citizen science initiatives. The articles are organized according to three themes.

## Theme I: Conceptual Relations Between Citizen Science and Social Innovation

Until recent years few papers emphasized the relation between citizen science and social innovation. In the presented collection, the team of Eckhardt et al., in their paper, goes further and points that ecosystem of co-creation is an essential feature of citizen science and introduces a form of collaborative scientific work with society. Included results from the H2020 SISCODE project show that co-creation is located inside and between various sectors of society. The subsequent study by Heinisch presents the role of translation in citizen science to foster social innovation. It examines the role of translation and terminology used in citizen science projects and how translation can support (or impede) social innovation through citizen science activities.

## Theme II: Learning Environments for Citizen Science and Social Innovation

The second part of this Research Topic contains contributions to studies focused on relations between teaching, learning, citizen science, and their potential relation to social innovation. The study by Roche et al. identifies challenges for successful integration of citizen science into mainstream education systems that also serve as signposts for possible synergies and opportunities. Another paper by Roche et al. continues the topic with a focus on Ireland’s rich history in public engagement with science. This study explores several aspects of citizen science in Ireland to assess its development and better understand potential opportunities for the field.

## Theme III: Methodological Issues in Usage and Development of Citizen Science and Social Innovation

The third theme opens the area to discuss methodological issues. It starts with the article of Goi and Tan, where the authors focus on methodological issues in using citizen science for the development of social innovations, in particular focusing on design thinking is an appropriate approach to be used by the community for future projects. Next, the article of Coulson et al. discusses citizen sensing as social innovation, where authors present data from their 2-year pan-European project. Finally, the paper by Scheibner et al. tackles ethical issues with using Internet of Things devices in citizen science studies.

## Conclusion

**FIGURE 1:**
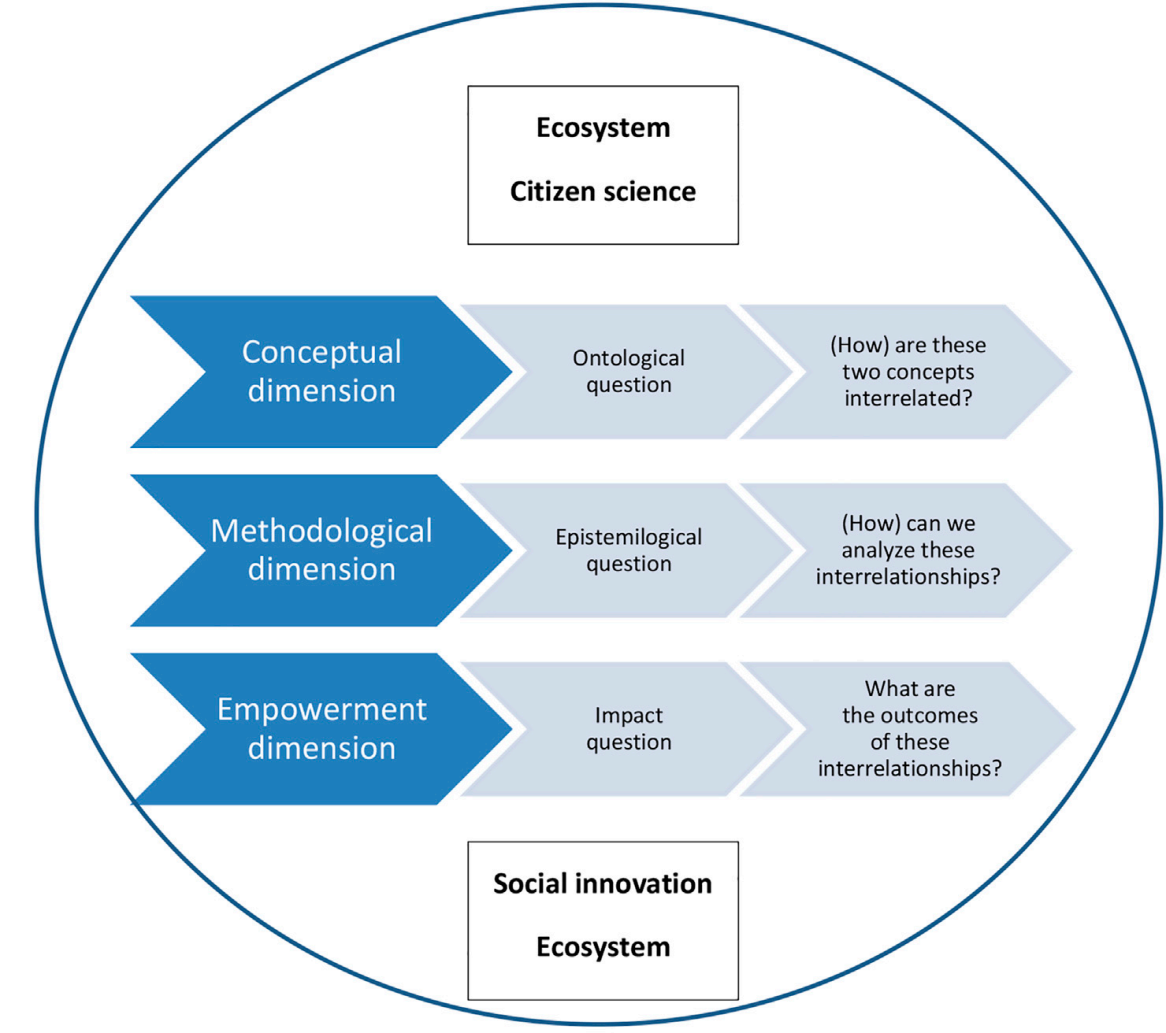
Analytical dimensions of relation between citizen science and social innovation. Source: own elaboration.

In this Research Topic, the editors wanted to open theoretical as well as empirically-based discussion, including examples, practices, and case studies of at least three types of relations between citizen science and social innovation: 1) domination of the citizen science features over social innovation aspects; 2) domination of the social innovation features over the citizen science aspects; and 3) the ways to achieve balance and integration between the social innovation and citizen science features. Each of these relationships highlights factors that influence the development of the primary scales of sustainability of innovations in the practice (Figure 1).

Moreover, the research results presented in the articles of this Research Topic allow the formulation of five directions for further research. These are: 1) dynamics of peer learning and organizational culture in citizen science and social innovation projects; 2) the personal capacity of social entrepreneurs, public managers, citizen scientists, and researchers; 3) design, evaluation, communication, and dissemination of results of the citizen science and social innovation initiatives; 4) digital social innovation and citizen science; and 5) co-creation and co-production processes and their impact on stakeholders (see also Schäfer and Kieslinger 2016; Anderson et al., 2020; Perelló et al., 2021). The editors hope this collection will be an inspiring introduction to studying both identified and yet unnoticed relations between citizen science and social innovation.
